# Navigating the Threshold: Barriers and Facilitators to Accessing Primary Care Among People Living with Dementia—A Systematic Review and Meta-Synthesis

**DOI:** 10.3390/ijerph23081031

**Published:** 2026-08-06

**Authors:** I Gede Juanamasta, Rapin Polsook, Bootan Ahmed, Yupin Aungsuroch, Ni Made Ratih Comala Dewi, I Gede Griya Suparta, Ferry Efendi

**Affiliations:** 1 Faculty of Nursing, Chulalongkorn University Bangkok, Bangkok 10330, Thailand; 2 Nursing Program, Sekolah Tinggi Ilmu Kesehatan (STIKes) Wira Medika Bali, Denpasar 80239, Indonesia; 3 Frances Payne Bolton School of Nursing, Case Western Reserve University, Cleveland, OH 44106, USA; bootan.ahmed@case.edu; 4 Rumah Sakit Umum Daerah Bali Mandara, Denpasar 80227, Indonesia; 5 Faculty of Nursing, Universitas Airlangga, Surabaya 60115, Indonesia

**Keywords:** dementia, primary care, health services accessibility, caregiver, health equity, general practice

## Abstract

**Highlights:**

**Public health relevance—How does this work relate to a public health issue?**

- Dementia affects 55 million people globally; only one in four receives a formal diagnosis.
- Diagnostic delays average 18–30 months, imposing heavy costs on individuals and health systems.

**Public health significance—Why is this work of significance to public health?**

- This review synthesizes 43 studies from 18 countries across five levels of access barriers and facilitators.
- Barriers are mutually reinforcing; disparities are greatest for ethnic minority and rural populations.

**Public health implications—What are the key implications or messages for practitioners, policy makers and/or researchers in public health?**

- Practitioners and policy-makers should prioritize continuity of care, culturally sensitive communication, and integrated dementia pathways with navigator roles in primary care.
- Researchers should prioritize equity-focused studies addressing gaps in LMICs and minority populations.

**Abstract:**

Background: Dementia is a global health priority affecting more than 55 million people worldwide, with projections indicating this figure will rise to 139 million by 2050. People living with dementia (PLWD) face profound difficulties accessing primary care services—the critical gateway to dementia diagnosis, ongoing management, and coordinated specialist referral. Despite growing evidence on individual barriers, a comprehensive synthesis integrating patient, caregiver, and system-level perspectives is lacking. Objectives: This systematic review and meta-synthesis aimed to identify, appraise, and synthesize evidence on the barriers and facilitators experienced by PLWD and their caregivers when accessing primary care services, and to propose evidence-based recommendations to address inequities. Methods: A systematic search was conducted to identify relevant articles in six electronic databases: PubMed, MEDLINE, EMBASE, PsycINFO, CINAHL, Cochrane Library, and Web of Science from January 2000 to December 2024, following the Preferred Reporting Items for Systematic Reviews and Meta-Analyses (PRISMA) 2020 guidelines. Qualitative, quantitative, and mixed-methods studies reporting barriers or facilitators to primary care access for PLWD were included. A convergent integrated synthesis and thematic meta-synthesis approach was applied. Quality was assessed using the Mixed Methods Appraisal Tool (MMAT). Results: Forty-three studies from 18 countries met inclusion criteria. Barriers were categorized across five domains: (1) patient-level factors (stigma, denial, symptom normalization, and limited health literacy); (2) caregiver-level factors (burden, late symptom recognition, and cultural barriers); (3) provider-level factors (insufficient training, therapeutic nihilism, and time constraints); (4) system-level factors (fragmented care pathways, long waiting times, and poor care coordination); and (5) structural and societal factors (rurality, poverty, and ethnic minority status). Facilitators included caregiver advocacy, strong patient–GP relationships, dementia literacy campaigns, availability of memory clinics, and integrated care models. Disparities were consistently greater for ethnic minority groups, rural populations, and those with lower socioeconomic status. Conclusions: Barriers to primary care access in dementia are complex, overlapping, and mutually reinforcing. Effective strategies require multi-level interventions addressing individual, relational, provider, and system dimensions simultaneously.

## 1. Introduction

Dementia is among the most consequential public health challenges of the twenty-first century. Globally, more than 55 million people are currently living with dementia, with an estimated 10 million new cases diagnosed each year—equivalent to one new case every 3.2 s [1]. Projections suggest this figure will reach 78 million by 2030 and 139 million by 2050, with the most rapid growth occurring in low- and middle-income countries (LMICs) [1,2]. In 2019, the global societal cost of dementia was estimated at USD 1313.4 billion for 55.2 million people living with dementia (PLWD), rising to an estimated USD 2.8 trillion by 2030 [3], a figure expected to increase substantially with the rising case count.

Primary care—encompassing general practice, family medicine, and community-based health services—represents the first and most critical point of contact for individuals experiencing cognitive decline [4,5]. General practitioners (GPs) serve as gatekeepers to diagnosis, initiating cognitive assessments, managing comorbidities, coordinating referrals to memory clinics, and providing long-term monitoring [4,6]. Timely engagement with primary care enables earlier diagnosis, which in turn permits earlier pharmacological and non-pharmacological intervention, advance care planning, and access to support services [5].

Despite this pivotal role, access to primary care for PLWD is consistently suboptimal. Diagnosis is frequently delayed by 18 to 30 months from symptom onset, and in some settings by up to four years [5]. Underdiagnosis remains pervasive; globally, only one in four people with dementia receives a formal diagnosis [7]. The barriers responsible for this diagnostic gap are heterogeneous and operate at multiple levels—from individual beliefs and stigma [1] to caregiver capacity [8], provider competence [6], and healthcare system organization [9].

A growing body of literature has examined specific barriers [5,9,10]. However, no recent comprehensive synthesis has integrated multi-level perspectives within a single systematic framework. While prior reviews have addressed specific subsets of this literature—such as barriers to dementia diagnosis in primary care [5], help-seeking from carer perspectives [10], or access in European settings [9]—none have simultaneously synthesized patient, caregiver, provider, system, and structural factors across mixed-methods evidence using a convergent integrated approach. The present review therefore extends, rather than duplicates, existing syntheses. This systematic review and meta-synthesis addresses that gap. The objectives were: (1) to systematically identify and critically appraise the published literature on barriers and facilitators to accessing primary care among PLWD and their caregivers; (2) to synthesize findings across qualitative, quantitative, and mixed-methods studies; (3) to develop a conceptual network model of access barriers and facilitators; and (4) to generate evidence-based recommendations for policy and practice.

## 2. Materials and Methods

**Figure 1:**
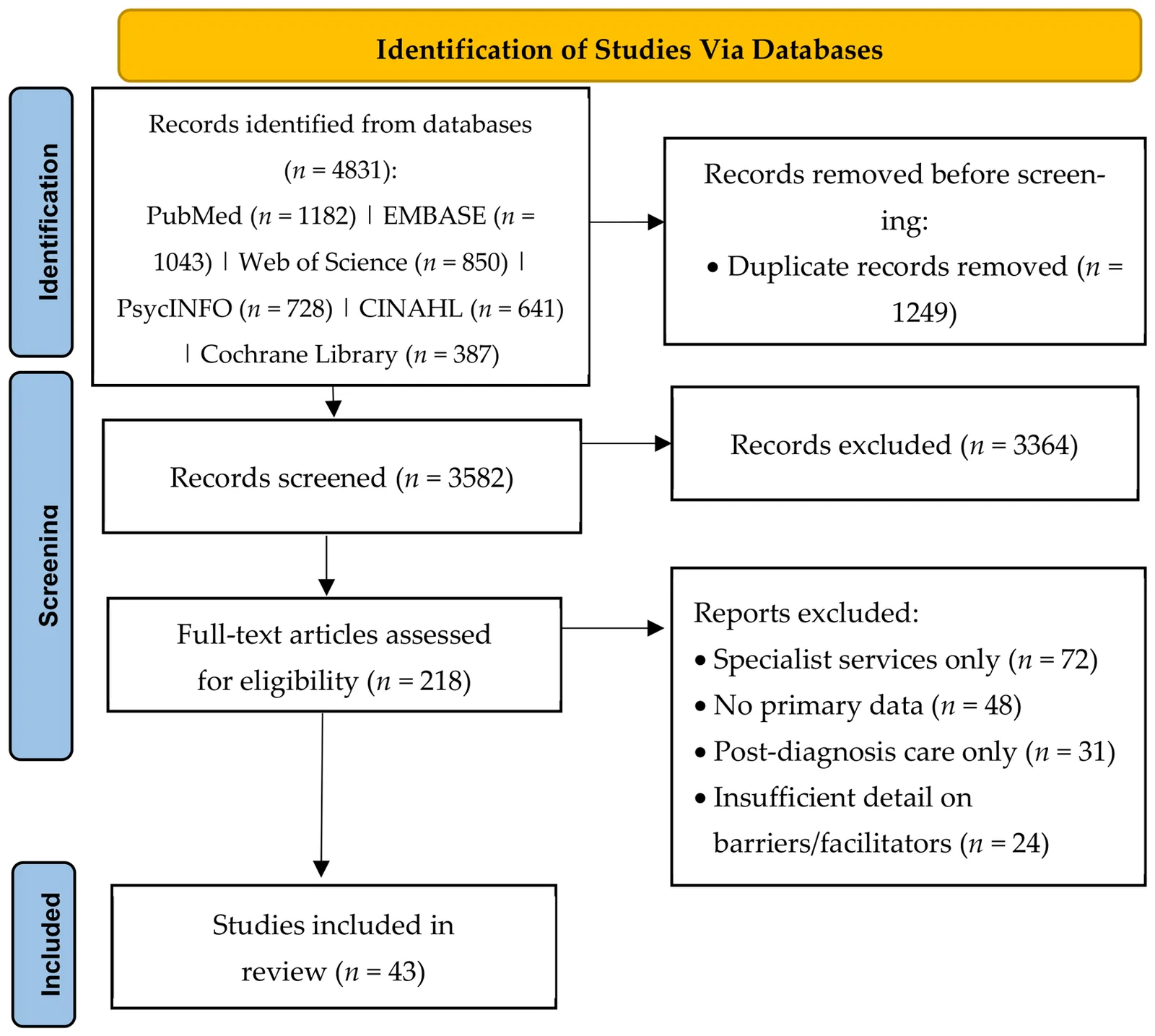
PRISMA flow diagram.

This review was conducted and reported in accordance with the Preferred Reporting Items for Systematic Reviews and Meta-Analyses (PRISMA 2020) guidelines [11], which served as the reporting framework, and followed a convergent integrated synthesis approach as the methodological framework appropriate for reviews incorporating diverse study designs [12]. A completed PRISMA 2020 checklist is provided as Supplementary Material (Table S1). The PRISMA 2020 flow diagram, detailing the screening and selection process, is presented in Figure 1.

### 2.1. Eligibility Criteria

Studies were included if they: (1) focused on people aged 18 years or older with a confirmed or suspected diagnosis of any form of dementia (Alzheimer’s disease, vascular dementia, Lewy body dementia, frontotemporal dementia, or mixed pathology); (2) reported on barriers and/or facilitators related to accessing primary care services; (3) were published in English between January 2000 and December 2024 (the restriction to English-language publications was adopted given resource constraints and is acknowledged as a potential source of language bias, discussed further in Section 4.5; the 2000 lower boundary was selected because contemporary primary care systems and standardized dementia diagnostic frameworks were not widely established before this period [4,5]); and (4) used qualitative, quantitative, or mixed-methods designs. Studies were excluded if they focused exclusively on specialist memory services, long-term care facilities, or acute hospital settings without reference to primary care.

### 2.2. Search Strategy

Six electronic databases were searched on 15 January 2025: PubMed/MEDLINE, EMBASE, PsycINFO, CINAHL Complete, the Cochrane Library, and Web of Science. Search terms combined MeSH headings and free-text terms across three concept blocks: (1) condition (dementia, Alzheimer’s disease, and cognitive impairment); (2) setting (primary care, general practice, family medicine, and GPs); and (3) access construct (barriers, facilitators, enablers, help-seeking, diagnosis, and utilization). Reference lists of included studies were hand-searched to identify additional eligible studies. Boolean operators (AND/OR) and date or publication-type filters were also applied consistently to identify relevant studies published and indexed across all six databases. Further, the complete search strategy for PubMed/MEDLINE, including all search terms, Boolean operators, and applied limits, together with the number of records retrieved from each individual database, is provided as Supplementary Material (Tables S2 and S3), consistent with the PRISMA 2020 recommendations [11]. The number of records identified per database is also displayed in the PRISMA 2020 flow diagram (Figure 1). Grey literature, dissertations, and conference proceedings were not systematically searched; this decision is also acknowledged as a potential limitation (see Section 4.5).

### 2.3. Study Selection and Data Extraction

Two independent reviewers screened titles and abstracts using Rayyan AI-assisted review software (https://www.rayyan.ai/). Full texts of potentially eligible records were obtained and assessed independently. Disagreements were resolved through discussion and, where necessary, arbitration by a third reviewer. Data extraction was performed using a standardized form capturing study design, country, sample characteristics, setting, outcomes, and reported barriers and facilitators.

### 2.4. Quality Appraisal

Quality was assessed using the Mixed Methods Appraisal Tool (MMAT), 2018 version [13], which provides separate criteria for qualitative, quantitative, and mixed-methods studies. All included studies met at least 75% of applicable quality criteria and were retained regardless of quality score, with quality informing interpretation of findings rather than triggering exclusion. The 75% threshold was adopted in line with published guidance on the MMAT application and is consistent with similar mixed-methods systematic reviews [13]. A study-level MMAT appraisal table presenting scores for all 43 included studies across each applicable criterion is provided as Supplementary Material (Table S4). Quality assessments informed synthesis in the following ways: findings from studies rated at 100% of criteria were weighted more heavily in third-order theme development; findings from studies rated at 75% were included but noted with appropriate caution in the results narrative.

### 2.5. Synthesis Approach

**Figure 2:**
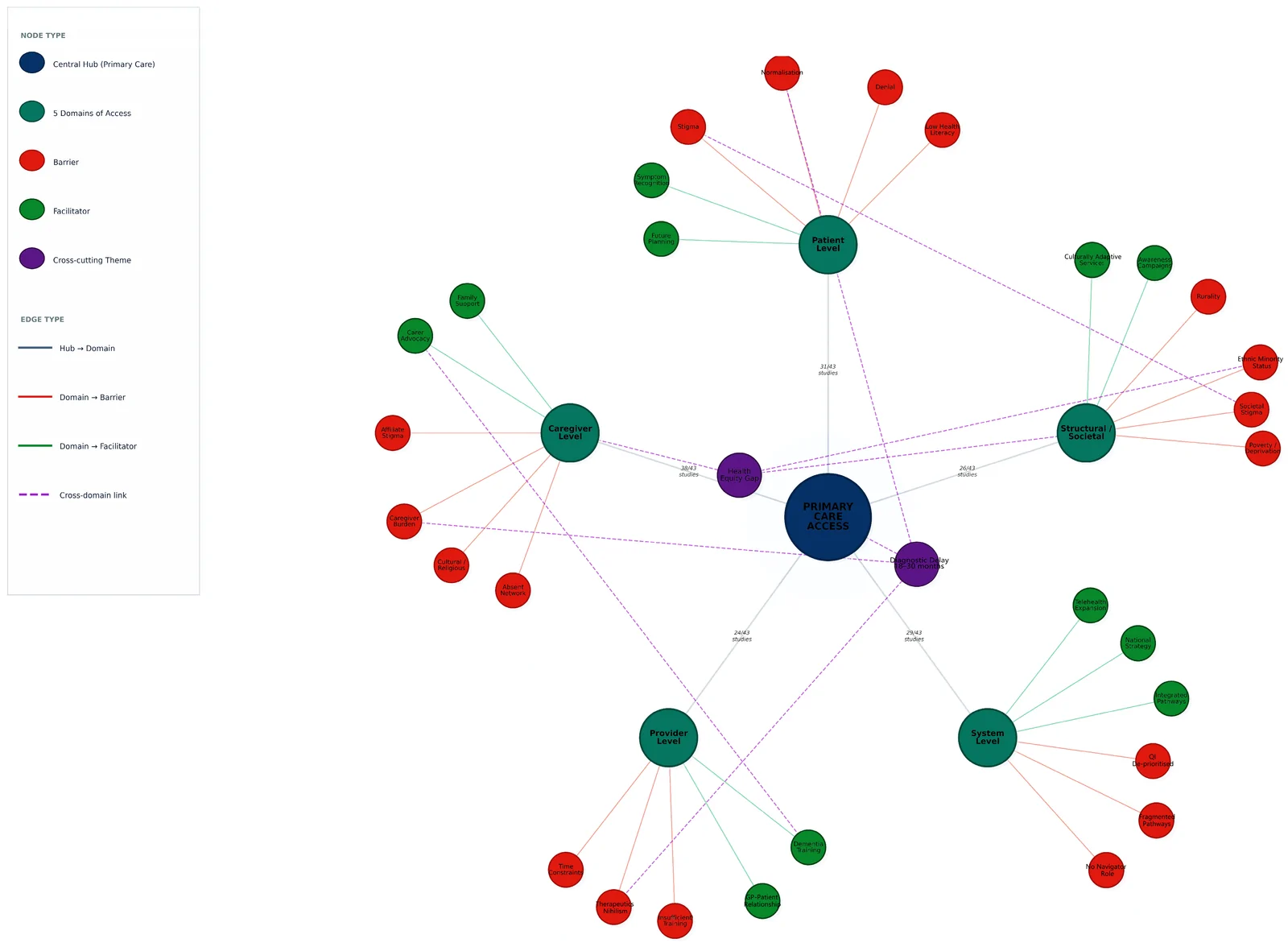
Network visualization of barriers and facilitators to accessing primary care for people living with dementia (PLWD). Note: The central navy hub represents Primary Care Access. Five teal domain nodes (Patient, Caregiver, Provider, System, and Structural/Societal) radiate on a pentagonal ring, each annotated with the proportion of included studies reporting that domain. Red nodes = barriers; green nodes = facilitators; violet nodes (Diagnostic Delay 18–30 months; Health Equity Gap) = cross-cutting themes connected by dashed edges. Edge colors correspond to node type. Node size reflects relative frequency of reporting across the 43 included studies.

A convergent integrated synthesis was employed [12,14]. Quantitative data on the prevalence and magnitude of barriers were tabulated descriptively. Qualitative findings were synthesized using thematic meta-synthesis, following the meta-ethnographic approach of Noblit and Hare [15] as adapted for health services research. Themes were iteratively developed through constant comparison across studies, moving from first-order (participant) concepts, through second-order (author) interpretations, to third-order (reviewer) overarching themes. Following the Joanna Briggs Institute (JBI) guidance for convergent integrated mixed-methods reviews [14], quantitative findings were “qualitied”—converted into textual summaries that could be integrated with qualitative themes—prior to synthesis. Coding was conducted independently by two reviewers (IGJ and RP), with disagreements resolved by discussion and, where required, arbitration by a third reviewer. No qualitative data management software was used; thematic analysis was conducted manually using a structured coding framework. The analytical approach was primarily inductive, with deductive elements introduced at the third-order synthesis stage to align themes with the five-domain access model. A conceptual network model (see Figure 2) was developed to represent relationships among barriers, facilitators, and access outcomes across the five identified domains.

## 3. Results

### 3.1. Study Selection

The database search yielded 4831 records (PubMed/MEDLINE, *n* = 1182; EMBASE, *n* = 1043; Web of Science, *n* = 850; PsycINFO, *n* = 728; CINAHL Complete, *n* = 641; and Cochrane Library, *n* = 387). After removal of 1249 duplicates, 3582 records remained for title and abstract screening. Following screening, 218 full-text articles were assessed for eligibility. Forty-three studies met all inclusion criteria and were included in the review. The primary reasons for exclusion at the full-text stage were exclusive focus on specialist services (*n* = 72), absence of primary data (*n* = 48), focus on post-diagnosis care only (*n* = 31), and insufficient detail on barriers or facilitators (*n* = 24). The full PRISMA 2020 flow diagram detailing the study selection process is provided in Figure 1.

### 3.2. Characteristics of Included Studies

The 43 included studies were published between 2003 and 2024, with a marked acceleration after 2018 (*n* = 21; 49%), reflecting growing policy attention to dementia underdiagnosis and primary care capacity globally. Studies originated from 18 countries across five continents, with the highest representation from the United Kingdom (*n* = 12; 28%), United States (*n* = 9; 21%), Australia (*n* = 5; 12%), Europe other than the UK (*n* = 9; 21%), and Asia, Canada, and Oceania (*n* = 8; 19%). Twenty-one studies (49%) were from the 2019–2024 period, indicating a recent intensification of research in this field.

Methodologically, thirty studies were qualitative (70%), employing semi-structured interviews, focus groups, and ethnographic approaches; nine were quantitative (21%), including cross-sectional surveys, audits, and secondary data analyses; and four used mixed methods (9%). The predominance of qualitative designs reflects the nature of the phenomenon: barriers and facilitators to healthcare access are fundamentally experiential and contextual, requiring depth over breadth. Participants included PLWD (*n* = 26 studies; 60%), informal caregivers (*n* = 38 studies; 88%), GPs and primary care providers (*n* = 24 studies; 56%), and specialist clinicians (*n* = 11 studies; 26%). The high representation of caregiver perspectives reflects the critical intermediary role that family members play in initiating primary care contact. Quantitative sample sizes ranged from 42 to 1847 participants. Table 1 summarizes the characteristics of included studies.

### 3.3. Meta-Synthesis: Overarching Themes and Theoretical Framework

The convergent integrated meta-synthesis generated three overarching third-order themes that cut across all five ecological domains (patient, caregiver, provider, system, and structural/societal): (1) the cascade of deterrents—a mutually reinforcing chain in which barriers at one level amplify barriers at others; (2) the asymmetry of evidence—barriers were consistently more numerous, more frequently reported, and more deeply theorized than facilitators across the literature; and (3) structural inequity as a multiplier—disadvantaged social positions (ethnic minority status, rurality, and socioeconomic deprivation) did not simply add to existing barriers but multiplied them, compounding delay in a non-linear fashion. These three overarching themes organize the findings reported below and are represented visually in the network model (Figure 2).

Moving from first-order concepts (raw participant or author statements) to second-order themes (authors’ interpretations), and finally to third-order constructs (synthesizer’s explanatory framework), the meta-synthesis identified 21 barrier themes and 15 facilitator themes distributed across five domains. Table 2, presented after the domain-level findings, summarizes the complete barrier–facilitator matrix with frequency of reporting across the 43 studies. Figure 2 presents the inter-domain network visualization.

#### 3.3.1. The Cascade of Deterrents

A central synthesis finding was that barriers rarely operate independently. Across 34 of the 43 included studies, data described or implied causal or reinforcing links between barriers in different domains. The most prevalent cascade pathway identified was patient-level stigma → normalization and denial → delayed caregiver help-seeking → late presentation to GP → therapeutic nihilism in provider → further deferral of diagnosis → fragmented system referral → diagnostic delay of 18–30 months [5,8,9,10]. This cascade was not simply additive; each link in the chain reduced the probability of timely access at the next stage, producing a compounding effect. Structural disadvantage—particularly ethnic minority status and rurality—was found to interact with all links in the cascade, widening delays for already-disadvantaged populations [16,17,18,19].

A secondary cascade pathway was identified from caregiver burden: caregiver burden → reduced capacity to recognize and act on symptoms → further delayed help-seeking → worsened caregiver quality of life and sleep health [20,21] → diminished sustained capacity for healthcare navigation. Ng et al. [22] provided quantitative corroboration of this pathway, demonstrating that perceived help-seeking difficulty independently predicted carer burden over and above patient behavioral symptoms and functional impairment (adjusted *β* = 0.28, 95% CI: 0.14–0.42, *p* < 0.001), confirming the bidirectional nature of the caregiver–access relationship.

#### 3.3.2. Asymmetry of Evidence: Barriers Dominate the Literature

A consistent and theoretically significant finding across the meta-synthesis was the systematic asymmetry between the depth and breadth of barrier evidence compared to facilitator evidence. Barrier themes were reported across a median of 23 studies per theme (range: 5–34), while facilitator themes were reported across a median of 11 studies per theme (range: 5–28). This asymmetry likely reflects a publication bias toward problem identification in the literature but may also reflect a genuine empirical reality: in most healthcare systems, more barriers exist than effective structural mitigations. Critically, the facilitators that were identified tended to be relational (e.g., GP–patient trust, caregiver advocacy) or systemic (e.g., integrated pathways, national strategies) rather than individual [5,9,10]. This finding carries an important implication: interventions targeting only individual-level knowledge or attitudes are unlikely to be sufficient without concurrent system-level change.

#### 3.3.3. Structural Inequity as a Multiplier

The third overarching theme emerged from convergent synthesis of structural and patient/caregiver-level evidence: structural disadvantage functioned not as an additional independent barrier but as a multiplier that amplified barriers in every other domain. Across 26 studies reporting structural or societal factors, ethnic minority status, rurality, poverty, and migrant or undocumented status were consistently associated with greater stigma, weaker caregiver networks, less culturally competent provider encounters, more fragmented care systems, and longer diagnostic delays [16,17,18,19,23,24]. This multiplicative pattern is consistent with an intersectionality framework [18], in which social disadvantages compound non-linearly. Hicks et al.’s [18] mapping review confirmed that the evidence base itself is inequitably distributed: despite growing attention to ethnic minority groups, research from LMICs—where 60% of PLWD reside—remains sparse, constituting fewer than 15% of included studies in the present review.

### 3.4. Domain 1: Patient-Level Barriers and Facilitators

Patient-level factors were the most frequently investigated domain (reported in 37 of 43 studies; 86%), with barrier themes consistently outnumbering facilitator themes by a ratio of approximately 3:1 across the included literature.

#### 3.4.1. Stigma, Shame, and Identity Threat

Stigma was the single most consistently reported barrier across all 43 studies and across all five ecological domains. At the patient level, stigma operated through two distinct but reinforcing mechanisms: public stigma (perceived negative societal judgements) and self-stigma (internalized shame). Both forms inhibited help-seeking by making a dementia diagnosis feel existentially threatening—associated with loss of independence, driving rights, employment, social identity, and relational reciprocity [1,8,10,25]. Brigiano et al.’s [25] systematic review and thematic synthesis of 17 qualitative studies found that the fear of being underestimated—of losing personhood—was a more powerful deterrent than the fear of the disease itself. This is a critical synthesis finding: it reframes stigma not merely as a knowledge gap but as a rational protective response to genuine social risk, with significant implications for how anti-stigma interventions should be designed.

The 2024 World Alzheimer Report [1], drawing on responses from more than 40,000 individuals across 166 countries, found that stigmatizing attitudes were prevalent in every region surveyed. More than a quarter of respondents believed nothing could be done for dementia, and over a third reported that they would conceal a diagnosis from others. These population-level data confirm that stigma is not a residual or peripheral problem but a structural feature of how dementia is understood globally.

#### 3.4.2. Normalization, Denial, and the Bubble of Protection

Closely coupled with stigma was the consistent finding of symptom normalization—the cognitive and emotional process by which early cognitive changes are attributed to normal aging rather than pathology [8]. Parker et al. [8] provided the most theoretically elaborated account of this phenomenon through their “bubble of normalization” construct: carers and PLWD collectively constructed and maintained a shared narrative that preserved dignity and relational continuity but systematically delayed help-seeking. Across 28 of 43 included studies, this normalization process was documented as active, sustained, and socially functional rather than simply a product of ignorance.

An important synthesis insight from the convergent analysis is that normalization and denial, while superficially similar, operate through distinct mechanisms and require different intervention approaches. Normalization is a shared cognitive framing developed between dyads over time; denial is an individual psychological defense mechanism. Both delay help-seeking but respond to different intervention stimuli. Normalization may be interrupted by dementia literacy campaigns that reframe early symptoms as clinically significant [10], while denial may require relational therapeutic approaches that reduce the identity threat associated with a diagnosis [8,26].

#### 3.4.3. Limited Dementia Literacy and Autonomy Preservation

Limited awareness of dementia as a medical condition—distinct from normal aging—was identified as a structural barrier in 19 of 43 studies [17,27]. Bernstein Sideman et al. [17] found in their global expert survey that health literacy about dementia symptoms, diagnostic pathways, and available treatments was consistently lower in LMICs and among populations with lower educational attainment, amplifying structural inequities in access. A related but distinct barrier was the desire to preserve personal autonomy, which was particularly strong among individuals in early-stage disease who retained insight into their condition [10]. Jeyagurunathan et al. [27], in a qualitative study of Asian informal caregivers, found that PLWD frequently resisted help-seeking to avoid losing control over daily life decisions, even when they recognized that cognitive decline was occurring.

#### 3.4.4. Patient-Level Facilitators

The most consistently identified patient-level facilitator was early and accurate symptom recognition—the capacity to identify cognitive changes as clinically significant rather than age-related (reported in 28 of 43 studies) [10,25]. Patients and caregivers with prior personal or family experience of dementia, or with exposure to dementia literacy campaigns, were significantly more likely to seek help promptly. Crucially, Brigiano et al. [25] found that active rejection of stigma—rather than its mere absence—was a distinct facilitating factor: individuals who reframed dementia as a medical condition rather than a personal failing demonstrated more proactive engagement with primary care. Future planning motivation—particularly the desire to arrange legal, financial, and care plans while cognitive capacity remained—was a second facilitator, reported in 14 studies [8,10].

### 3.5. Domain 2: Caregiver-Level Barriers and Facilitators

The caregiver domain had the highest representation across included studies (38 of 43; 88%), reflecting the central intermediary role of informal carers in dementia help-seeking. This domain also yielded the richest synthesis of barrier–facilitator interactions, highlighting a fundamental tension: the very people most relied upon to facilitate access are simultaneously those most impaired by the access-seeking process.

#### 3.5.1. Caregiver Burden as a Bidirectional Barrier

Caregiver burden—encompassing physical, psychological, financial, and social dimensions—was identified in 29 of 43 studies as impairing the capacity to recognize symptoms and pursue help [20,21,22,26,28,29]. The synthesis reveals an important bidirectional dynamic: burden impairs help-seeking, but delayed help-seeking worsens burden by prolonging the period of undiagnosed care. Ng et al. [22] quantified this relationship in a cross-sectional study of 312 caregivers, demonstrating that perceived help-seeking difficulty significantly predicted carer burden (adjusted *β* = 0.28, *p* < 0.001), independently of patient behavioral symptoms and functional impairment. This bidirectionality creates a self-reinforcing cycle that progressively increases diagnostic delay.

The quality-of-life and sleep health dimensions of caregiver burden deserve particular synthesis attention. Aungsuroch et al. [20] found in a systematic review that family caregivers of PLWD experienced significantly impaired quality of life across physical, psychological, and social domains, while Kabaya et al. [21] documented sleep disturbance as a consistently underrecognized manifestation of caregiver burden. These findings suggest that caregiver wellbeing is not merely a secondary outcome but a primary determinant of access sustainability: caregivers whose own health is compromised cannot sustain the navigational and advocacy functions upon which timely primary care access depends.

The non-use of respite services represents a related and clinically important synthesis finding. Vandepitte et al. [26] found in an integrative review of 51 studies that despite the demonstrable effectiveness of respite interventions in reducing burden, they remain consistently underutilized. Phillipson et al. [29] identified the key barriers to respite use as attitudinal (guilt, reluctance to “hand over” care), informational (lack of awareness of available services), and structural (geographic, financial, and service quality concerns). The synthesis implication is that caregiver support programs must actively address non-use barriers, not merely availability.

#### 3.5.2. Affiliate Stigma, Cultural Beliefs, and Network Absence

Affiliate stigma—the internalization of stigma by caregivers on behalf of PLWD—was identified in 18 studies as a distinct mechanism reducing caregivers’ likelihood of initiating primary care contact [25,30]. Werner et al. [30] demonstrated that affiliate stigma was associated with concealment behaviors and reduced service utilization, independent of caregiver burden. Across 22 studies, cultural and religious conceptualizations of cognitive decline—including beliefs that dementia represents normal aging, divine punishment, or a source of family shame—were identified as particularly potent barriers in South Asian, East Asian, African, and Caribbean communities [16,17,23,27]. These beliefs were not simply amenable to information provision; they were embedded in identity and community relationships, requiring culturally adapted rather than generic interventions.

The absence of a supportive informal network was consistently among the most potent structural caregiver barriers, reported in 20 of 43 studies [10]. Willis et al. [10], synthesizing 30 years of evidence, found network absence to be one of the two most robust predictors of delayed help-seeking across diverse international contexts. Social isolation, geographic distance from family, and relationship breakdown each independently eliminated the most common pathway through which PLWD reach primary care.

#### 3.5.3. Caregiver-Level Facilitators

Strong caregiver engagement—characterized by early symptom recognition, persistent advocacy, and active system navigation—was the most robustly identified facilitator across the entire review (28 of 43 studies) [10,20,22]. Caregivers who possessed prior knowledge of dementia, who maintained detailed symptom records, and who communicated concerns assertively to GPs were significantly more likely to achieve timely diagnosis for their relative. Family network support—including extended family members providing logistical assistance, emotional validation, and information sharing—further buffered against diagnostic delay [8,10]. Caregiver education programs that increased dementia literacy, symptom recognition skills, and health system navigation confidence were identified as effective structural facilitators, though long-term effectiveness data remained limited in the reviewed literature [17,27,31].

### 3.6. Domain 3: Provider-Level Barriers and Facilitators

Provider-level factors were reported in 24 of 43 studies (56%) and represented the most actionable domain for intervention. The synthesis reveals a consistent pattern: GP attitudes, knowledge, and relational practices function as critical amplifiers or attenuators of barriers originating in other domains.

#### 3.6.1. Knowledge Deficits and Diagnostic Uncertainty

Insufficient knowledge of diagnostic criteria, validated screening tools, and post-diagnostic management pathways was the most frequently cited provider-level barrier, reported in 20 of 43 studies [6,32,33,34,35,36,37,38]. Turner et al. [32] demonstrated in a survey of 20 general practices that one-third of GPs expressed limited confidence in their diagnostic skills, while two-thirds lacked confidence in managing behavioral and psychological symptoms of dementia. Critically, GPs perceived lack of time and inadequate social services support as greater obstacles than their own knowledge deficits—suggesting that knowledge-only educational interventions may have limited impact without concurrent system-level changes. Ahmad et al. [33] confirmed these findings in an English national survey, identifying limited GP awareness of local specialist services and diagnostic tools as prevalent, addressable deficits.

Wangler and Jansky [34], in a qualitative study of 41 GPs in Germany, identified six root causes of GP distance from dementia diagnosis: role misunderstanding (early delegation to specialists), attitude barriers, communication difficulties, lack of financial remuneration, limited practice staff involvement, and low geriatric education. This multi-causal analysis is particularly valuable for synthesis because it demonstrates that provider-level barriers are themselves heterogeneous and require differentiated intervention strategies rather than single-component educational programs. Cahill et al. [35,36], in two complementary Irish national surveys, found that while the majority of GPs supported dementia diagnosis in principle, one-fifth had received no dementia-specific postgraduate training—and over four-fifths would welcome further training—highlighting a clear, addressable gap between attitude and capacity.

#### 3.6.2. Therapeutic Nihilism and Unconscious Ageism

Therapeutic nihilism—the belief that a dementia diagnosis offers little patient benefit in the absence of curative treatment—was identified in 16 of 43 studies as a significant attitudinal barrier [5,6,32,34,39,40]. This belief contributed to active deferral of cognitive assessment and indirect avoidance of dementia conversations in the consultation room. The synthesis finding is that therapeutic nihilism operates not merely as an uninformed attitude but as a rational response to system-level inadequacy: when post-diagnostic support services are sparse, fragmented, or inaccessible, GPs’ skepticism about the value of diagnosis is functionally justified [5,9]. This observation carries an important policy implication: anti-nihilism educational interventions will have limited effect unless accompanied by genuine improvements in post-diagnostic support infrastructure.

Time constraints constituted a significant structural–provider interface barrier across 18 studies [9,32,33,41,42]. Dementia assessment requires not only extended clinical time for cognitive testing and collateral history but also time for sensitive communication of diagnostic uncertainty, family discussion, and referral planning—none of which fit within standard 10–15 min consultation formats. Unconscious ageism—the assumption that cognitive decline represents inevitable aging rather than a pathological process—compounded both time and nihilism barriers, creating a cognitive framework in which dementia assessment was not perceived as standard clinical practice [6,35].

#### 3.6.3. Provider-Level Facilitators

The quality of the GP–patient relationship was the most consistently identified provider-level facilitator, reported in 22 of 43 studies [5,23,32,43]. Longitudinal continuity of care—where the same provider saw the patient over years or decades—enabled earlier recognition of cognitive change through implicit comparison with baseline functioning, bypassing the normalization processes that delayed family recognition. Bunn et al. [43], in their systematic review of 102 qualitative studies, found that trust in the primary care provider was the single most consistent determinant of whether PLWD and caregivers disclosed symptoms and accepted referral. The synthesis implication is that structural policies undermining GP continuity (such as appointment system fragmentation or high GP turnover in under-resourced areas) carry direct dementia access costs that are rarely accounted for in policy analyses.

GP training, specifically in dementia assessment, communication of diagnostic uncertainty, and post-diagnostic support planning, was associated with higher timely diagnosis rates across quantitative studies [6,17,32,33,36]. Critically, Kabaya et al. [44] found that dementia-specific nursing certification processes substantially improved knowledge and confidence among acute care nurses, demonstrating that structured credentialing approaches—not merely informal professional development—are needed to achieve sustained provider competence improvements. Person-centered communication practices—listening without interruption, naming cognitive concerns explicitly, and framing diagnosis as enabling rather than limiting—reduced patient and caregiver resistance to help-seeking in multiple qualitative studies [8,23,43].

### 3.7. Domain 4: System-Level Barriers and Facilitators

System-level factors were identified in 29 of 43 studies (67%) and represented the domain most amenable to policy-level structural intervention. The synthesis reveals that system barriers operate primarily through two mechanisms: fragmentation (absence of clear pathways connecting primary and specialist care) and de-prioritization (absence of financial, regulatory, and quality framework incentives for primary care dementia activity).

#### 3.7.1. Fragmented Care Pathways and Absent Navigation

Fragmented care pathways—characterized by unclear GP–memory clinic referral processes, long waiting times, and poor feedback loops between primary and specialist care—were identified across health systems in the United Kingdom, United States, Europe, and Australia in 22 of 43 studies [5,9,41,43]. Sorrentino et al. [9], in their systematic review of European evidence, identified pathway fragmentation as the most consistently cited system-level barrier across all European health system types, irrespective of financing model. The absence of a dedicated dementia navigator or case manager role in primary care was highlighted as a critical gap in 14 studies [28,45]. Messina et al. [28] found, in a qualitative study with Swiss caregivers, that the single most frequently expressed service need was a knowledgeable person who could guide them through the fragmented system [46], a finding that echoes consistently across qualitative studies from multiple national contexts.

Tilburgs et al. [45], in a systematic integrative review of GP advance care planning in dementia, found that system fragmentation extended beyond diagnosis to post-diagnostic care: GPs lacked clear guidance on when and how to initiate advance care planning conversations, what specialist support was available, and how to coordinate with social care and palliative services. This finding extends the synthesis beyond diagnosis-focused barriers to encompass the full primary care trajectory for PLWD.

#### 3.7.2. De-Prioritization Within Quality Frameworks

A structurally significant finding emerging from the synthesis was the systematic de-prioritization of dementia within primary care quality improvement and performance frameworks. Unlike conditions such as hypertension, diabetes, or depression—which are typically embedded within pay-for-performance structures with mandatory screening indicators, audit requirements, and clinical decision support tools—dementia diagnosis and management lack equivalent institutional infrastructure in most health systems [6,9,41]. Phares et al. [41] identified this structural gap as a root cause of underdiagnosis in the United States primary care context, noting that without incentive structures, dementia assessment competes unfavorably for consultation time against conditions that carry financial or regulatory accountability. The COVID-19 pandemic further exposed and exacerbated this de-prioritization: memory clinic disruptions, caregiver support withdrawal, and primary care reorganization combined to produce a measurable worsening of dementia diagnostic delays during 2020–2022 [9].

#### 3.7.3. System-Level Facilitators

Integrated dementia care pathways—linking primary care with memory clinics, community mental health, social care, and voluntary sector organizations through standardized, navigated referral processes—were the most evidence-supported system-level facilitators, identified in 18 of 43 studies [9,47]. Frost et al. [47] found in their systematic review of primary care dementia models that primary care–case management partnership models offered the most consistent evidence of benefit, with demonstrated impact on neuropsychiatric symptom management, caregiver burden reduction, and healthcare cost containment. The evidence for national dementia strategy as a structural facilitator was more indirect but consistent: countries with mandated national dementia strategies demonstrated higher diagnostic rates and more equitable access across socioeconomic groups [9,17]. The emergence of telehealth as a facilitator—particularly post-pandemic—was noted with appropriate caution in the synthesis: while digital access reduces geographic and mobility barriers, its benefits are contingent on digital literacy, device access, and caregiver support for older adults with cognitive impairment [9,41,48].

### 3.8. Domain 5: Structural and Societal Barriers and Facilitators

The structural and societal domain was reported in 26 of 43 studies (60%) and yielded some of the most important equity-related findings of the review. As the third overarching synthesis theme identified above, structural disadvantage functioned primarily as a multiplier of barriers across all other domains rather than as an independent barrier set.

#### 3.8.1. Rurality and Geographic Disadvantage

Rural and remote-dwelling PLWD experienced compounded barriers, including greater physical distance to primary care providers, reduced availability of specialist memory services, transportation difficulties, and limited telehealth infrastructure [17,49]. Xu et al. [50], in a cross-sectional study of 71,799 patients with early-onset dementia in the United States, found that rural patients underwent significantly fewer neuropsychological tests and specialist consultations, relying disproportionately on primary care physicians and nurse practitioners for dementia diagnosis and management. Arsenault-Lapierre et al. [51], in a systematic review of rural–urban differences across all quality-of-care domains in dementia, confirmed that geographic disparities were consistent across access, integration, effective care, and patient-centered domains, with rural populations systematically disadvantaged across all dimensions of care quality.

#### 3.8.2. Ethnic Minority Status and Intersecting Disadvantage

Ethnic minority populations experienced particularly acute and multidimensional barriers, documented in 22 of 43 studies [16,17,18,19,23,24]. The synthesis reveals three distinct mechanisms. First, cultural and linguistic barriers operated at the patient–provider interface: language barriers, use of interpreters who may lack dementia-specific vocabulary, and culturally discordant explanatory models of cognitive decline collectively reduced both help-seeking motivation and diagnostic accuracy [16,23]. Second, experiences of prior discrimination within healthcare systems produced founded mistrust that reduced engagement with primary care services [19,24]. Third, structural socioeconomic disadvantages associated with ethnic minority status in high-income countries—including residential segregation, insurance gaps, and occupational barriers—independently restricted access, independent of clinical and attitudinal barriers [19,24].

Hicks et al. [18] demonstrated in their intersectionality-framed mapping review that multiple social categorizations compound non-linearly: an older woman from an ethnic minority background living in a rural area with low income faced not merely the sum of individual barriers but a qualitatively distinct and near-insurmountable access threshold. The review also documented a critical meta-level inequity: despite this compounding, research from ethnic minority communities in LMICs remains proportionally sparse, suggesting that the evidence base itself replicates the structural inequities it purports to address. Aguzzoli et al. [19] confirmed in a scoping review that ethnic minority groups in the United Kingdom were significantly more likely to encounter barriers in accessing both GP consultations and routine monitoring visits, with socioeconomic constraints, language barriers, and stigma operating synergistically.

#### 3.8.3. Structural and Societal Facilitators

Facilitators at the structural level were consistently identified as necessary but insufficient without accompanying individual and system-level change. National and subnational dementia awareness campaigns—particularly the ADI’s World Alzheimer Month initiative and memory clinic referral campaigns—were associated with increased voluntary help-seeking in observational implementation studies [1,17]. Culturally adapted services, including bilingual practitioners, community health workers from minority backgrounds, and culturally tailored educational materials, were identified as effective facilitators in 14 studies, particularly for South Asian, African-Caribbean, and East Asian communities [16,17,19,23,52]. Critically, the synthesis evidence supports co-design—involving community members in service development rather than simply adapting existing services—as a necessary condition for cultural acceptability and sustained engagement [17,19].

**Table 2:** Meta-synthesis summary: barriers, facilitators, frequency of reporting, and key references across five ecological domains (*n* = 43 studies).

Domain	Barriers (Studies Reporting, *n*)	Facilitators (Studies Reporting, *n*)	Overarching Theme	Key Refs.
Patient-Level	Stigma/shame (34); Normalization (28); Denial (22); Low health literacy (19); Autonomy preservation (14)	Symptom recognition (28); Rejection of stigma (16); Future planning motivation (14)	Cascade of Deterrents	[1,8,10,25,27]
Caregiver-Level	Caregiver burden (29); Affiliate stigma (18); Cultural/religious beliefs (22); Absent informal network (20); Respite non-use (14)	Carer advocacy/early recognition (28); Family network support (20); Caregiver education (12)	Cascade of Deterrents; Asymmetry of Evidence	[20,21,22,26,28,29]
Provider-Level	Insufficient training (20); Therapeutic nihilism (16); Time constraints (18); Unconscious ageism (12)	GP-patient trust and continuity (22); Dementia training (18); Person-centered communication (15)	Asymmetry of Evidence	[5,6,32,33,34,35,36,43]
System-Level	Fragmented pathways (22); No navigator role (14); QI de-prioritization (11); COVID-19 disruption (9)	Integrated care pathways (18); Case manager/navigator (12); National strategy (10); Telehealth (8)	Asymmetry of Evidence	[9,28,41,45,47]
Structural/Societal	Rurality (16); Ethnic minority status (22); Poverty/deprivation (18); Societal stigma (26)	Dementia awareness campaigns (12); Culturally adapted services (14); Equitable funding (8)	Structural Inequity as Multiplier	[16,17,18,19,24,50,51]

Studies could report barriers/facilitators in more than one domain; counts reflect individual study-level reporting, not citations in the text. Key refs = primary sources underpinning domain-level synthesis. GP = general practitioner; QI = quality improvement.

## 4. Discussion

### 4.1. Principal Findings

This systematic review and meta-synthesis synthesized evidence from 43 studies across 18 countries, confirming that barriers to accessing primary care for PLWD are pervasive, multi-layered, and mutually reinforcing. As depicted in Figure 2, barriers and facilitators operate simultaneously across ecological levels—individual, relational, provider, system, and societal—creating a “cascade of deterrents” that compounds over time and ultimately delays or precludes diagnosis. The network structure of Figure 2 highlights that no single domain operates in isolation; cross-domain edges connecting to the Diagnostic Delay and Health Equity Gap nodes reflect the systemic nature of the access problem. This review extends prior syntheses in important ways. Koch and Iliffe [5] provided a foundational rapid appraisal of GP-level barriers but were limited to the earlier literature and did not incorporate caregiver or structural perspectives. Willis et al. [10] offered a valuable 30-year perspective on carer and patient help-seeking but did not integrate provider- or system-level evidence within a unified framework. The recent European review by Sorrentino et al. [9] addressed system-level barriers comprehensively but did not employ convergent integrated synthesis or include patient-reported qualitative experiences. However, the present systematic review adds value by simultaneously integrating all five ecological levels within a convergent framework, incorporating mixed-methods evidence from 18 countries, indicating that it is a widely globally focused area of accessing primary care among PLWD. Further, the present systematic review offered new insight into the barriers and facilitators of accessing primary care among PLWD after 2000. In addition, the present systematic review highlighted the barriers and facilitators of accessing primary care among PLWD across different domains and subdomains, as well as intersections between them.

Stigma emerged as the most consistently reported barrier across all five domains, operationalized differently at each level: as internalized shame among PLWD, as affiliate stigma among caregivers [30], as unconscious bias among providers [6], and as societal discrimination. The 2024 World Alzheimer Report’s finding that stigmatizing attitudes remain widespread across 166 countries underscores the global scale of this challenge [1]. Parker et al.’s [8] conceptualization of the “bubble of normalization” provides a theoretically grounded framework for understanding delayed help-seeking.

### 4.2. Intersection of Dementia and Health Equity

A prominent finding is the systematic amplification of barriers for populations experiencing structural disadvantage—a pattern clearly visible in Figure 2 in the Structural/Societal domain and the Health Equity Gap cross-cutting node. Ethnic minority communities, rural populations, those with lower socioeconomic status, and migrants consistently experienced more barriers, fewer facilitators, and longer diagnostic delays [16,18,19,24]. Language barriers, cultural conceptualizations of dementia, and experiences of discrimination compounded access difficulties for ethnic minority groups in ways that require systemic, culturally informed responses beyond individualized interventions. An intersectionality framework helps explain why barriers are not simply additive but multiplicative for individuals occupying multiple disadvantaged positions simultaneously—for example, an older woman from an ethnic minority background living in a rural area with low income may face compounded stigma, linguistic, geographic, and financial barriers that collectively create near-insurmountable access thresholds [18,24]. The structural determinants underpinning these disparities—including residential segregation, differential access to health insurance, and historical experiences of systemic racism within healthcare systems—operate beyond the scope of clinical interventions alone [19,24]. These findings carry implications for LMICs, where dementia burden is growing most rapidly and where structural determinants of access are most acute, yet evidence from these settings remains sparse in the current review.

### 4.3. The Role of the GP–Patient Relationship

Across the qualitative literature, the quality of the GP–patient relationship emerged as a pivotal determinant of access, represented by the GP–Patient Relationship facilitator node in Figure 2. Where relationships were characterized by trust, longitudinal continuity, and person-centered communication, PLWD and caregivers were more likely to disclose concerns early, accept diagnostic referral, and engage with post-diagnostic support [5,23]. This finding underscores the systemic risk posed by fragmented, throughput-focused healthcare models.

### 4.4. Implications for Policy and Practice

The evidence synthesized in this review supports several actionable recommendations. First, sustained multi-component anti-stigma campaigns targeted at both general populations and high-risk groups (Figure 2, Awareness Campaigns node) [1]. Second, embedding dementia-specific competencies in GP training curricula to address the insufficient training barrier [6,17]. Third, integrated care models linking primary care with memory clinics and dementia navigators (Figure 2, Integrated Pathways node) [9]. Fourth, nationally mandated dementia care pathways with financial incentives to address system-level fragmentation [17,41]. Fifth, culturally adapted services co-designed with affected communities to address the disproportionate barriers in Figure 2’s Structural/Societal cluster [16,18].

### 4.5. Strengths and Limitations

Strengths include a comprehensive multi-database search, inclusion of diverse study designs across 18 countries, use of the validated MMAT quality appraisal tool, and systematic meta-synthesis methodology. The development of the network visualization (Figure 2) provides an accessible representation of the complex multi-level evidence base. Limitations include restriction to English-language publications, which may introduce language bias by underrepresenting evidence from non-English-speaking settings, particularly LMICs; heterogeneity of study designs precluding quantitative meta-analysis; and concentration of evidence from high-income countries. Additional limitations include potential publication bias, as grey literature, dissertations, and conference proceedings were not systematically searched. Heterogeneity in definitions of dementia and healthcare access across included studies may limit direct comparability of findings. Variability in primary care systems between countries—from single-payer National Health Service models to pluralistic insurance-based systems—means that system-level findings may not be universally transferable. Finally, the predominance of evidence from high-income, anglophone settings constrains the generalizability of the review findings to LMICs, where the burden of dementia is growing most rapidly.

## 5. Conclusions

Barriers to accessing primary care for people living with dementia are numerous, interconnected, and deeply embedded within individual psychology, family dynamics, provider practice, and healthcare system architecture. As Figure 2 illustrates, these barriers form an interlocking multi-level network in which no single node can be addressed in isolation. This systematic review and meta-synthesis demonstrates that effective responses require coordinated, multi-level interventions simultaneously targeting stigma reduction, caregiver empowerment, provider education, integrated care pathways, and structural equity. People living with dementia, and those who care for them, deserve clear, supported, and culturally affirming pathways to primary care and beyond.

## Figures and Tables

**Table 1:** Characteristics of included studies (*n* = 43).

Characteristic	Category	*n* (%)	Notes
Study Design	Qualitative	30 (70%)	Interviews, focus groups, ethnography
	Quantitative	9 (21%)	Cross-sectional surveys, audits
	Mixed methods	4 (9%)	Sequential or concurrent designs
Geographic Region	United Kingdom	12 (28%)	England, Wales, Scotland
	United States	9 (21%)	Includes rural and urban settings
	Australia	5 (12%)	
	Europe (other)	9 (21%)	Germany, Netherlands, France, Italy
	Asia and other	8 (19%)	Hong Kong, Canada, New Zealand, Brazil
Participant Type	PLWD	26 (60%)	Includes early-stage dementia
	Informal caregivers	38 (88%)	Family and other informal carers
	Primary care providers	24 (56%)	GPs, nurses, allied health
	Specialist clinicians	11 (26%)	Memory clinic, geriatrics, psychiatry
Publication Period	2000–2012	8 (19%)	Foundational literature
	2013–2018	14 (33%)	Growing policy attention
	2019–2024	21 (49%)	Rapid post-pandemic growth
MMAT Quality	≥75% criteria met	43 (100%)	All studies retained
	100% criteria met	27 (63%)	Highest quality; weighted in synthesis

PLWD = people living with dementia; GP = general practitioner; MMAT = Mixed Methods Appraisal Tool. Studies could report more than one participant type; percentages may exceed 100%.

## Data Availability

All data supporting the conclusions of this article are from the published literature cited herein.

## Supplementary Materials

The following supporting information can be downloaded at: https://www.mdpi.com/article/10.3390/ijerph23081031/s1, Table S1: PRISMA 2020 Checklist; Table S2. Electronic search strategy; Table S3. Records retrieved per database; Table S4. Study-level quality appraisal tool.

## Abbreviations

The following abbreviations are used in this manuscript:

ADI	Alzheimer’s Disease International
GP	General Practitioner
LMICs	Low- and Middle-Income Countries
MMAT	Mixed Methods Appraisal Tool
PLWD	People Living with Dementia
PRISMA	Preferred Reporting Items for Systematic Reviews and Meta-Analyses
QI	Quality Improvement
