# Supplementary material for: Navigating the Threshold: Barriers and Facilitators to Accessing Primary Care Among People Living with Dementia—A Systematic Review and Meta-Synthesis
Source: Int J Environ Res Public Health. 2026 Aug 6;23(8):1031. doi: 10.3390/ijerph23081031 (PMC13513581; doi:10.3390/ijerph23081031)
Supplement: Supplementary file 1 [file ijerph-23-01031-s001.zip › ijerph-4335164-supplementary.pdf]

Supplementary Table S1. PRISMA 2020 Checklist

Manuscript title: Navigating the Threshold: Barriers and Facilitators to Accessing Primary Care Among People Living with Dementia—A Systematic Review and Meta-Synthesis

Journal: International Journal of Environmental Research and Public Health

Note: Page numbers refer to the revised manuscript. N/A = not applicable to this review. This checklist was completed in accordance with Page MJ et al. The PRISMA 2020 statement: an updated guideline for reporting systematic reviews. BMJ 2021;372:n71[11].

Item #	Checklist item	Location in manuscript	Notes / Comments
**TITLE**			
1	Identify the report as a systematic review.	p.1	
**ABSTRACT**			
2	See PRISMA 2020 for Abstracts checklist.	p.2	
**INTRODUCTION**			
3	Rationale: Describe the rationale for the review in the context of existing knowledge.	p.2-3	
4	Objectives: Provide an explicit statement of the objective(s) or question(s) the review addresses.	p.2-3	
**METHODS**			
5	Eligibility criteria: Specify the inclusion and exclusion criteria for the review.	p.4	
6	Information sources: Specify all databases, registers, websites, organisations, reference lists and other sources searched or consulted.	p.4	
7	Search strategy: Present the full search strategies for all databases, registers and websites, including any filters and limits used.	p.4	
8	Selection process: Specify the methods used to decide whether a study met the inclusion criteria, including how many reviewers screened each record and resolved disagreements.	p.4	
9	Data collection process: Specify the methods used to collect data from reports, including how many reviewers collected data from each report, and any processes for obtaining or confirming data from study investigators.	p.4	
10a	Data items: List and define all outcomes for which data were sought.	p.4	
10b	List and define all other variables for which data were sought.	p.4	
11	Study risk of bias assessment: Specify the methods used to assess risk of bias in the included studies.	p.4-5	
12	Effect measures: Specify for each outcome the effect measure(s) used in the synthesis or presentation of results.	N/A	
13a	Synthesis methods: Describe the processes used to decide which studies were eligible for each synthesis.	p.5	
13b	Describe any methods required to prepare the data for presentation or synthesis.	p.5	

Item #	Checklist item	Location in manuscript	Notes / Comments
13c	Describe any methods used to tabulate or visually display results of individual studies and syntheses.	p.4	
13d	Describe any methods used to synthesize results (including measures of consistency).	p.4	
13e	Describe any methods used to explore possible causes of heterogeneity among study results.	p.4	
13f	Describe any sensitivity analyses conducted to assess robustness of the synthesized results.	N/A	
14	Reporting bias assessment: Describe any methods used to assess risk of bias due to missing results in a synthesis.	N/A	
15	Certainty assessment: Describe any methods used to assess certainty (or confidence) in the body of evidence.	N/A	
**RESULTS**			
16a	Study selection: Describe the results of the search and selection process, including reasons for exclusions at each stage of the process. Use a flow diagram.	p.3; Figure 1	
16b	Cite studies that might appear to meet the inclusion criteria, but which were excluded, and explain why they were excluded.	p.6	
17	Study characteristics: Cite each included study and present its characteristics.	p.7; Table 1	
18	Risk of bias in studies: Present assessments of risk of bias for each included study.	Supp. S4	
19	Results of individual studies: For all outcomes, present, for each study: (a) summary statistics for each group (where appropriate) and (b) an effect estimate and its precision.	p.7–15	
20a	Results of syntheses: For each synthesis, briefly summarise the characteristics and risk of bias among contributing studies.	p.7–15	
20b	Present results of all statistical syntheses conducted.	p.7–15	
20c	Present results of all investigations of possible causes of heterogeneity among study results.	N/A	
20d	Present results of all sensitivity analyses conducted to assess the robustness of the synthesized results.	N/A	
21	Reporting biases: Present assessments of risk of bias due to missing results for each synthesis assessed.	N/A	
22	Certainty of evidence: Present assessments of certainty (or confidence) in the body of evidence for each outcome assessed.	p.7–15	
**DISCUSSION**			
23a	Discussion: Provide a general interpretation of the results in the context of other evidence.	p.7–9	
23b	Discuss any limitations of the evidence included in the review.	p.9	
23c	Discuss any limitations of the review processes used.	p.9	

Item #	Checklist item	Location in manuscript	Notes / Comments
23d	Discuss implications of the results for practice, policy, and future research.	p.8–9	
**OTHER INFORMATION**			
24a	Registration: Provide registration information for the review, including register name and registration number, or state that the review was not registered.	Not registered	
24b	Indicate where the review protocol can be accessed, or state that a protocol was not prepared.	Supp. File	
24c	Describe and explain any amendments to information provided at registration or in the protocol.	N/A	
25	Support: Describe sources of financial or other support for the review, and the role of the funders or sponsors in the review.	p.18	
26	Competing interests: Declare any competing interests of review authors.	p.18	
27	Availability of data, code and other materials: Report which of the following are publicly available and where they can be found.	p.18	

Table S2. Electronic search strategy.

The PubMed/MEDLINE search strategy is presented below, executed on 15 January 2025. The strategy combined Medical Subject Headings (MeSH) and free-text (title/abstract, [tiab]) terms across three concept blocks—condition, setting, and access construct—combined with the Boolean operator AND. Equivalent strategies were adapted for EMBASE (Emtree headings), PsycINFO and CINAHL Complete (subject headings via the EBSCOhost platform), the Cochrane Library (CENTRAL), and Web of Science (Core Collection, topic search), with controlled-vocabulary terms translated to each database's indexing system. Filters were applied for English language and publication date (1 January 2000 to 31 December 2024).

#	Concept Block	Search Terms (PubMed/MEDLINE syntax)
1	**Condition**	`"Dementia"[Mesh] OR "Alzheimer Disease"[Mesh] OR dementia[tiab] OR alzheimer*[tiab] OR "cognitive impairment"[tiab] OR "cognitive decline"[tiab]`
2	**Setting**	`"Primary Health Care"[Mesh] OR "General Practice"[Mesh] OR "primary care"[tiab] OR "general practi*"[tiab] OR "family medicine"[tiab] OR GP[tiab]`
3	**Access**	`"Health Services Accessibility"[Mesh] OR barrier*[tiab] OR facilitator*[tiab] OR "help-seeking"[tiab] OR access*[tiab] OR diagnos*[tiab]`
4	**Combined + limits**	`#1 AND #2 AND #3; Filters: English[lang], 2000/01/01-2024/12/31[Date - Publication]`

Note: [Mesh] = Medical Subject Heading; [tiab] = title/abstract field; asterisk (*) = truncation wildcard. Within each concept block, MeSH and free-text terms are combined with OR; the three blocks are combined with AND. The final search yielded 1,182 records from PubMed/MEDLINE prior to deduplication.

Table S3. Records retrieved per database.

Database (platform)	Records identified (n)
PubMed/MEDLINE	1,182
EMBASE (Ovid)	1,043
Web of Science (Core Collection)	850
PsycINFO (EBSCOhost)	728
CINAHL Complete (EBSCOhost)	641
Cochrane Library (CENTRAL)	387
**Total records identified**	**4,831**

After removal of 1,249 duplicate records, 3,582 unique records proceeded to title and abstract screening (see PRISMA 2020 flow diagram, Figure 1).

Table S4. Study-level quality appraisal tool (MMAT, version 2018).

All 43 included studies were appraised with the MMAT 2018 [13]. Each study was assessed against two screening questions (S1, S2) applicable to all designs, followed by the five criteria specific to its methodological category (qualitative, n = 30; quantitative, n = 9; mixed-methods, n = 4). Studies were retained regardless of score; quality informed the weighting of findings during synthesis. All included studies met at least 75% (≥ 4/5) of applicable criteria; 27 of 43 (63%) met 100% (5/5).

Table S4a. Qualitative studies (n = 30) — MMAT criteria 1.1–1.5

Study (first author, year) [ref]	S1	S2	1.1	1.2	1.3	1.4	1.5	Criteria met
Iliffe et al., 2009 [4]	●	●	●	●	●	●	●	**5/5 (100%)**
Raphael, 2022 [6]	●	●	●	●	●	●	●	**5/5 (100%)**
Koch & Iliffe, 2010 [5]	●	●	●	●	●	●	●	**5/5 (100%)**
Parker et al., 2020 [10]	●	●	●	●	●	●	●	**5/5 (100%)**
Parker et al., 2022 [8]	●	●	●	●	●	●	●	**5/5 (100%)**
Messina et al., 2022 [28]	●	●	●	●	●	●	●	**5/5 (100%)**
Kornder et al., 2025 [23]	●	●	●	●	●	●	●	**5/5 (100%)**
Kabaya et al., 2024 [44]	●	●	●	●	●	●	●	**5/5 (100%)**
Brigiano et al., 2025 [25]	●	●	●	●	●	●	●	**5/5 (100%)**
Jeyagurunathan et al., 2024 [27]	●	●	●	●	●	●	●	**5/5 (100%)**
Wangler & Jansky, 2020 [34]	●	●	●	●	●	●	●	**5/5 (100%)**
Bunn et al., 2012 [43]	●	●	●	●	●	●	●	**5/5 (100%)**
Tilburgs et al., 2018 [45]	●	●	●	●	●	●	●	**5/5 (100%)**
Gibson et al., 2024 [37]	●	●	●	●	●	●	●	**5/5 (100%)**
Wollney et al., 2022 [39]	●	●	●	●	●	●	●	**5/5 (100%)**
Chen & Lin, 2022 [31]	●	●	●	●	●	●	●	**5/5 (100%)**
Beck et al., 2021 [46]	●	●	●	●	●	●	●	**5/5 (100%)**
Abe et al., 2021 [38]	●	●	●	●	●	●	●	**5/5 (100%)**
Ahmed et al., 2024 [49]	●	●	●	●	●	●	●	**5/5 (100%)**
Phillips et al., 2012 [40]	●	●	●	●	●	●	●	**5/5 (100%)**
Aguzzoli et al., 2026 [19]	●	●	●	●	●	●	◐	**4/5 (80%)**
Aungsuroch et al., 2024 [20]	●	●	●	●	●	◐	●	**4/5 (80%)**
Kabaya et al., 2023 [21]	●	●	●	●	◐	●	●	**4/5 (80%)**
Leocadie et al., 2018 [26]	●	●	●	●	●	●	◐	**4/5 (80%)**
Phillipson et al., 2013 [29]	●	●	●	●	●	◐	●	**4/5 (80%)**

Study (first author, year) [ref]	S1	S2	1.1	1.2	1.3	1.4	1.5	Criteria met
Frost et al., 2020 [47]	●	●	●	◐	●	●	●	**4/5 (80%)**
Arsenault-Lapierre et al., 2023 [51]	●	●	●	●	◐	●	●	**4/5 (80%)**
Hicks et al., 2025 [18]	●	●	●	●	●	●	◐	**4/5 (80%)**
Jones et al., 2024 [42]	●	●	●	●	●	◐	●	**4/5 (80%)**
Jenkins & Kamal, 2024 [52]	●	●	●	●	◐	●	●	**4/5 (80%)**

Criteria: 1.1 qualitative approach appropriate to the research question; 1.2 data collection methods adequate; 1.3 findings adequately derived from the data; 1.4 interpretation sufficiently substantiated by data; 1.5 coherence between data sources, collection, analysis, and interpretation.

Table S4b. Quantitative studies (n = 9) — MMAT criteria 4.1–4.5 (quantitative descriptive)

Study (first author, year) [ref]	S1	S2	4.1	4.2	4.3	4.4	4.5	Criteria met
Ng et al., 2021 [22]	●	●	●	●	●	●	●	**5/5 (100%)**
Werner et al., 2012 [30]	●	●	●	●	●	●	●	**5/5 (100%)**
Turner et al., 2004 [32]	●	●	●	●	●	●	●	**5/5 (100%)**
Ahmad et al., 2010 [33]	●	●	●	●	●	●	●	**5/5 (100%)**
Xu et al., 2022 [50]	●	●	●	●	●	●	●	**5/5 (100%)**
Cahill et al., 2006 [35]	●	●	●	●	●	◐	●	**4/5 (80%)**
Cahill et al., 2008 [36]	●	●	●	●	◐	●	●	**4/5 (80%)**
Phares et al., 2026 [41]	●	●	●	◐	●	●	●	**4/5 (80%)**
Balls-Berry & Babulal, 2022 [24]	●	●	●	●	●	●	◐	**4/5 (80%)**

Criteria: 4.1 sampling strategy relevant to the research question; 4.2 sample representative of the target population; 4.3 measurements appropriate; 4.4 risk of nonresponse bias low; 4.5 statistical analysis appropriate.

Table S4c. Mixed-methods studies (n = 4) — MMAT criteria 5.1–5.5

Study (first author, year) [ref]	S1	S2	5.1	5.2	5.3	5.4	5.5	Criteria met
Giebel et al., 2021 [16]	●	●	●	●	●	●	●	**5/5 (100%)**
Bernstein Sideman et al., 2022 [17]	●	●	●	●	●	●	●	**5/5 (100%)**
Sorrentino et al., 2025 [9]	●	●	●	◐	●	●	●	**4/5 (80%)**
Liang & Aranda, 2023 [48]	●	●	●	●	◐	●	●	**4/5 (80%)**

Criteria: 5.1 adequate rationale for a mixed-methods design; 5.2 components effectively integrated; 5.3 outputs of integration adequately interpreted; 5.4 divergences/inconsistencies addressed; 5.5 components adhere to the quality criteria of each tradition involved.

Legend and notes

● = criterion met (Yes) ◐ = cannot tell / unclear ○ = criterion not met (No)

S1 = Are there clear research questions? S2 = Do the collected data allow the research questions to be addressed? Overall criteria met is reported as a count and percentage of the five design-specific criteria, in line with common MMAT reporting practice; the MMAT developers discourage reliance on a single overall score, and percentages are presented here only to summarise the pattern of methodological strengths and limitations.
